# Letter to the editor, international urology and nephrology—in silico–in vitro–in vivo: can numerical simulations based on computational fluid dynamics (CFD) replace studies of the urinary tract?

**DOI:** 10.1007/s11255-021-02869-9

**Published:** 2021-04-29

**Authors:** W. Kram, N. Buchholz

**Affiliations:** 1 Deptartment of Urology, University Medical Center Rostock, Schillingallee 35, 18057 Rostock, Germany; 2 U-Merge Research Office, Athens, Greece

Editor,

We read with interest the paper by Tal Amitay-Rosen et al. [1]*.*

Numerical simulations based on computational fluid dynamics (CFD) were used to examine the degree of renal fluid pressure build-up and patterns of urine flow in a stented ureter in the presence of obstructions to evaluate conditions leading to stent failure. Obstructions were examined both, in the form of closure within the ureter lumen due to the physical effect of extrinsic ureteral obstruction (EUO), and to occlusion within the stent lumen due to mineral encrustation, either separately or simultaneously. The effects of up to 100% obstruction, in the stent and ureter lumina and/or the side holes, were compared.

The CFD simulations showed that stent failure under EUO tends to occur suddenly and only when both, ureter and stent lumina, are almost completely blocked.

Based on this CDF simulation, the authors attempt to describe complex physiological conditions in the renal pelvis and ureter with only a few parameters leading to stent failure.

The following parameters for Newtonian fluid are accepted:

- Laminar flow,
- Incompressible fluid.

Not considered were:

- Dilatation of the ureter after obstruction and recent manipulation/ stent insertion;
- Differences in elastic wall properties in different parts of the ureter;
- Effects of peristalsis;
- Vesicoureteral reflux;
- Viscosity of urine and urinary sedimentation.

The authors noted that none of the reported CDF simulations has considered the effects of occlusions on renal fluid pressure. There are of course good reasons for this. With respect to urodynamic studies of the upper urinary tract, stented and obstructed ureters show a variety of pathological responses that cannot be fully characterized with the simulation presented here.

- Acute ureteral intubation causes an elevation of intrarenal pressure which is directly correlatated to the stent diameter. The intrarenal pressure decreases back to baseline after 3 weeks [2]. Increased intrarenal reflux is noted in up to 90% of patients following stent placement for acute ureteral obstruction [3, 4] which also decreases back to baseline. Peristalsis gets diminished and usually recurs after about 8 weeks [5].

The specific pathological changes that occur during upper tract obstruction depend on the nature of the obstruction, and particularly whether it is acute or chronic in nature.

- Acute obstruction results in an increase in hydrostatic intrarenal pressure and leads to stretching of the renal capsule and the ureteral wall. The frequency of the peristaltic waves increases, whilst their amplitude decreases. The degree of obstruction and the time of its development play an important role. Initially, there is an increase of intrarenal pressure and intrarenal blood perfusion, followed within up to 4 h by a decrease [6–8].
- In extrinsic ureteral obstruction, i.e., due to a tumor or pregnancy (EUO), the obstruction usually develops gradually and is not comparable to an acute obstruction, i.e., by a ureteral stone. The increased pressure in the proximal tubule and at Bowman’s capsule leads to a decrease in glomerular filtration rate. Renal blood flow decreases, eventually leading to ischemia and chronic renal failure [9].

For all those reasons, CFD simulation is probably not suitable for describing the flow-oriented physiological conditions of the upper urinary tract. However, it may be suitable to simulate pressure-based closure mechanisms, i.e., in the urinary bladder.
